# Machine learning-based identification of tumor-infiltrating immune cell-associated model with appealing implications in improving prognosis and immunotherapy response in bladder cancer patients

**DOI:** 10.3389/fimmu.2023.1171420

**Published:** 2023-03-31

**Authors:** Hualin Chen, Wenjie Yang, Zhigang Ji

**Affiliations:** Department of Urology, Peking Union Medical College Hospital, Chinese Academy of Medical Sciences and Peking Union Medical College, Beijing, China

**Keywords:** immunotherapy, bladder cancer, immune checkpoint, immune infiltration, prognosis, machine learning

## Abstract

**Background:**

Immune cells are crucial components of the tumor microenvironment (TME) and regulate cancer cell development. Nevertheless, the clinical implications of immune cell infiltration-related mRNAs for bladder cancer (BCa) are still unclear.

**Methods:**

A 10-fold cross-validation framework with 101 combinations of 10 machine-learning algorithms was employed to develop a consensus immune cell infiltration-related signature (IRS). The predictive performance of IRS in terms of prognosis and immunotherapy was comprehensively evaluated.

**Results:**

The IRS demonstrated high accuracy and stable performance in prognosis prediction across multiple datasets including TCGA-BLCA, eight independent GEO datasets, our in-house cohort (PUMCH_Uro), and thirteen immune checkpoint inhibitors (ICIs) cohorts. Additionally, IRS was superior to traditional clinicopathological features (e.g., stage and grade) and 94 published signatures. Furthermore, IRS was an independent risk factor for overall survival in TCGA-BLCA and several GEO datasets, and for recurrence-free survival in PUMCH_Uro. In the PUMCH_Uro cohort, patients in the high-IRS group were characterized by upregulated CD8A and PD-L1 and TME of inflamed and immunosuppressive phenotypes. As predicted, these patients should benefit from ICI therapy and chemotherapy. Furthermore, in the ICI cohorts, the high-IRS group was related to a favorable prognosis and responders have dramatically higher IRS compared to non-responders.

**Conclusions:**

Generally, these indicators suggested the promising application of IRS in urological practices for the early identification of high-risk patients and potential candidates for ICI application to prolong the survival of individual BCa patients.

## 1 Introduction

Bladder cancer (BCa) is one of the most common urological cancers worldwide, with growing prevalence and mortality (1–3). During the past several decades, the therapeutic paradigm of BCa has shifted from traditional surgery to a multidisciplinary approach including surgery, chemotherapy, radiotherapy, and immunotherapy (4, 5). Evolution in treatment strategies benefits BCa patients in terms of overall survival (OS) and recurrence-free survival (RFS). However, not all BCa patients showed satisfactory responses to these treatments, especially chemotherapy, and immunotherapy. For non-response patients, not only have to suffer adverse effects and heavy medical costs but also, more importantly, they may fail to receive additional therapy for their progressed disease (6).

BCa, especially the advanced stage, is characterized by heterogeneity. It has been largely documented that tumoral heterogeneity is responsible for distinct response rates to chemotherapy and immune checkpoint inhibitors (ICI) treatment. Therefore, a robust prediction biomarker is urgently needed to address this concern. Recently, several candidate biomarkers such as programmed death-ligand 1 (PD-L1) expression and tumor mutation burden (TMB) have been tested for their performances in the clinical selection of candidates for immunotherapy. However, limited accuracy and intratumoral heterogeneity may challenge the wide application of these biomarkers (7). Therefore, it is imperative to identify robust and reliable biomarkers to facilitate the selection of patients for ICI treatment and improve prognosis, in the present personalized medicine era. Considering the inter- and intra-tumoral heterogeneity in BCa, a multigene signature holding homogenous expression profiles, may be an attractive method to address this challenge. Based on the advance in bioinformatics, numerous prognostic multigene signatures have been developed (8, 9). But these signatures are seldom used in clinical practices because of moderate accuracy, low reliability, and unsuitable machine learning methods.

Tumor cells, stromal cells, immune cells, and non-cellular molecules compose the intricate tumor microenvironment (TME). Previous studies have reported the critical role of immune cells in the regulation of tumor progression, metastasis, and resistance to therapy (10). Thus, deciphering the tumor immune microenvironment can boost our understanding of the molecular mechanisms and identify promising therapeutic targets.

In the study, we developed an immune cell infiltration-based mRNA signature and verified the performance in multiple public datasets and an in-house cohort (PUMCH-Uro). Our findings may facilitate the early identification of high-risk BCa patients, direct the application of ICI treatment, and further help BCa patients benefit from survival.

## 2 Methods

### 2.1 BCa transcriptomic datasets

To develop a robust prognostic signature, BCa RNA-seq datasets with complete survival data were obtained from the GDC portal of TCGA and GEO databases: TCGA-BLCA (n = 399, normal samples were discarded), GSE32894 (n = 224), GSE13507 (n = 165), GSE32548 (n = 128), GSE31684 (n = 93), GSE48075 (n = 73), GSE48277 (n = 71), GSE70691 (n = 49), GSE69795 (n = 38). TCGA-BLCA was used for signature development and other GEO datasets were for the validation. Clinical traits of TCGA-BLCA and GEO datasets were procured from cBioPortal and original papers, respectively. Details of survival information and clinical phenotypes of all datasets were summarized in **Table S1**.

### 2.2 ICI RNA-seq cohorts

To verify the predictive performance of the signature, pretreatment samples with complete transcriptomic data and clinical details from 13 ICI RNA-Seq cohorts were systemically collected. A total of 1010 patients were obtained and the corresponding transcriptomic data were integrated and batch effects were adjusted by Combat in the sva R package (11). Details of all datasets were summarized in **Table S2**.

### 2.3 Immune cell infiltration

28 immune cell-related signatures were obtained from the study of Charoentong et al. (12) (**Table S3**). Single-sample Gene Set Enrichment Analysis (ssGSEA) of the GSVA R package was used to quantify enrichment scores for each pairing of a gene set and sample.

Besides, other six methodologies including TIMER, xCell, MCPcounter, ESTIMATE, EPIC, and quanTIseq were used to assess the immune cell infiltration abundances. These analyses were performed by R package IOBR (13).

### 2.4 Consensus clustering

Based on the infiltration levels of 28 immune cells, k-means method-based unsupervised consensus clustering was performed to identify distinct immune cell infiltration-based molecular clusters of the TCGA-BLCA dataset. To guarantee the stability of clustering, a total of 1,000 times repetitions were performed using the R package ConsensusClusterPlus (14). Cumulative distribution function (CDF) curves and proportion of ambiguous clustering (PAC) scores were collectively used to decide the optimal number of clustering, as stated in the study by Liu et al. (15).

### 2.5 Weighted gene co-expression network analysis

WGCNA has been widely used in bioinformatics applications to identify modules of highly correlated genes and relate modules to sample traits (e.g., survival, stages, and grades). This method facilitated correlation network-based candidate biomarkers or therapeutic target selection. The analytic process was employed according to the formal package instructions (https://horvath.genetics.ucla.edu/html/CoexpressionNetwork/Rpackages/WGCNA/). In brief, the input data was a matrix of 399 TCGA-BLCA samples (after outlier removal) with complete traits and 5000 genes with top median absolute deviation. Then, we performed the automatic, one-step network construction, and module detection. The correlations between modules and traits were analyzed by Pearson’s correlation test and visualized by a “module-trait” heatmap. The most significant module with the highest correlation and p < 0.05 was regarded as the hub module. Correspondingly, module genes with gene significance > 0.5 & module membership > 0.6 were trait-related ones and selected for further analysis. Data analyses and visualization were employed by the WGCNA package (16).

### 2.6 Machine learning

Before machine learning, univariate Cox regression analysis was performed to identify prognosis-related genes. Subsequently, we integrated a total of 101 combinations based on 10 machine learning algorithms (stepwise Cox, random survival forest [RSF], elastic network [Enet], supervised principal components [SuperPC], partial least squares regression for Cox [plsRcox], CoxBoost, survival support vector machine [survival-SVM], Lasso, Ridge, and generalized boosted regression modeling [GBM]) based on 10-fold cross-validation to fit immune cell infiltration-related signatures (IRS) in TCGA-BLCA. Harrell’s concordance index (C-index) was subsequently calculated in eight independent GEO datasets to select an optimal IRS with the highest C-index. The details of model construction and parameter tuning are described in the **Supplementary Methods**.

### 2.7 BCa tissue samples and RNA sequencing

164 primary BCa tissue samples from patients with histologically diagnosed urothelial carcinoma were obtained from the Peking Union Medical College Hospital (PUMCH), Peking, China. All samples were formalin-fixed and paraffin-embedded (FFPE) for sequencing and IHC. BCa tumors were staged and graded according to the standard criteria (17). Baseline clinical traits were retrieved from the electronic medical record system. After the removal of samples with incomplete baseline clinical traits and/or follow-up data, 113 eligible ones were included in the cohort (PUMCH_Uro). Written informed consent was obtained from each patient. The study design was approved by the Institutional Review Board of PUHMH.

Total RNA was harvested using Trizol reagent (Sigma-Aldrich, USA) and purified with Monarch Total RNA Miniprep Kit (NEB, USA). RNA purity and RNA integrity were assessed using the NanoDrop ND-1000 spectrophotometer (Thermo Scientific, USA) and the Agilent 2100 Bioanalyzer (Agilent Technologies, USA), respectively. A total amount of 1 µg RNA per sample was used as input material for the RNA sample preparations. The sequencing libraries were constructed using NEBNext UltraTM RNA Library Prep Kit (NEB, USA) following the manufacturer’s protocol, and index codes were added to attribute sequences to each sample. PCR amplification was performed and the biotin-labeled probe was used for capturing target regions after the removal of the PCR primer. The library preparations were sequenced on an Illumina Hiseq platform and 125 bp/150 bp paired-end reads were generated. The adaptor sequences and low-quality sequence reads were removed from the raw reads. After data processing, raw reads were transformed into clean reads. Clean reads per sample were then mapped to the reference genome sequence (GRCh38/hg38 assembly) using bowtie2. FeatureCounts v2.0.1 was then used to count the number of reads mapped to each gene. The TPM of each gene was calculated based on the gene length and read count.

### 2.8 Immunohistochemistry

FFPE tissue from the PUMCH_Uro cohort was used for the IHC assay as previously reported (18). Paraffin sections were first incubated with primary antibodies against CD8A (Proteintech, China) and PD-L1 (Proteintech, China) and then a goat peroxidase-conjugated secondary antibody (Proteintech, China). After washing, the sections were stained with DAB and hematoxylin for signal visualization.

### 2.9 Statistical analysis

Statistical analyses and visualization were conducted using R v4.1.3 (https://www.r-project.org). Pearson correlation was employed to evaluate the association between IRS and T exhausted (TEX) signature and expressions of CD8A and PD-L1. Chi-squared test and Student’s t-test were employed to compare categorical and continuous variables, respectively. To assess the performance of IRS across datasets, the receiver operating characteristic (ROC) curves were used. For survival variables, the time-dependent ROC curves were used. The area under the ROC curve (AUC) quantitatively indicated the predictive performance. The Kaplan-Meier curve and log-rank test were performed to compare survival differences. Statistical significance was set two-tail p-value < 0.05.

## 3 Results

### 3.1 Consensus clustering stratified BCa TME into two subclusters with distinct immunological landscapes

**Figure 1:**
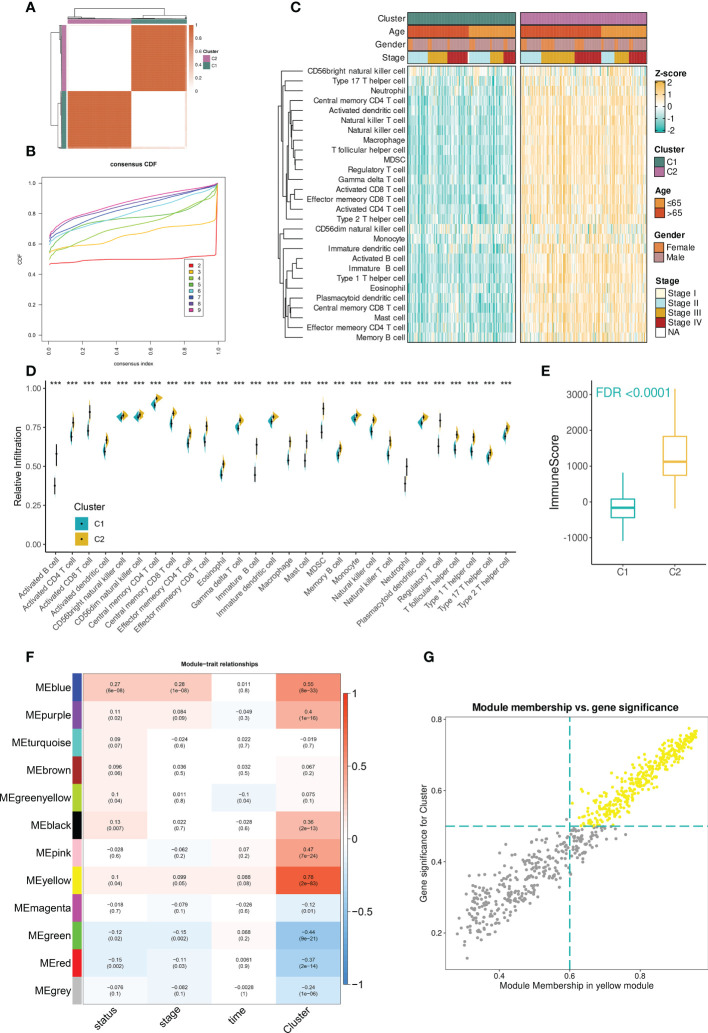
Identification of immune cell infiltration-related genes in TCGA-BLCA dataset. **(A)** The consensus score matrix when the number of clustering was 2. The score between two samples was positively correlated with the sample’s similarity which decided whether the two samples could be classified into the same cluster. **(B)** The CDF curves of consensus matrix. Each color indicated each number of clustering. **(C)** The differences in infiltration profiles of 28 immune cells between two clusters. The infiltration profiles were evaluated by ssGSEA and then normalized and scaled into Z-score. ComplexHeatmap package was used to produce this heatmap. **(D)** The infiltration levels of 28 immune cells between two clusters (***P < 0.001). **(E)** The distribution of immune score between two clusters. The immune score was evaluated by the ESTIMATE algorithm provided by the IOBR package. FDR, an FDR-adjusted p-value. **(F)** The “module-trait” heatmap presented the correlations between each module and each trait. Within a cell (block), the number above and below represented the correlation coefficient and p-value, respectively. The red/blue color of the cell indicated a positive/negative correlation, and the color ranging from white to red or blue indicated the correlation increased gradually. **(G)** Scatterplot demonstrated the correlation between GS and MM in the yellow module (r = 0.97). Each dot was a gene and these dots in yellow color represented ones satisfying the threshold (MM > 0.6 & GS > 0.5).

The workflow of the study was illustrated in **Figure S1**. In the TCGA-BLCA dataset, the infiltration abundances of 28 immune cells per sample were calculated by ssGSEA. Based on the infiltration profiles, we conducted the unsupervised consensus clustering and stratified BCa TME into two subclusters (**Figure 1A**). CDF curves and PAC scores determined the optimal number of clustering (k = 2, **Figures 1B**, **S2A**). Deconvolution of TME uncovered that 28 immune cells were more highly infiltrated in the TME of cluster 2 compared to that of cluster 1 (**Figures 1C, D**). Hence, cluster 2 was related to the inflamed TME whereas cluster 1 dominated the non-inflamed. To verify the stability of clustering and the differences in immunological phenotypes, we further decoded TME contexture using other five methodologies, including TIMER, xCell, MCPcounter, ESTIMATE, EPIC, and quanTIseq. **Figure 1E** showed significantly higher immune scores of cluster 2 when compared to cluster 1. Similar findings were illustrated in **Figure S2B**.

### 3.2 Identification of immune cell infiltration-related genes

Next, to facilitate immune cell infiltration-related gene identification, we constructed the correlation networks by WGCNA. Under the selected R^2^ of 0.85, the optimal soft threshold was set to 5 (**Figure S2C**). The cluster dendrogram showed that the input 5000 genes were clustered into twelve modules based on their expression similarities (**Figure S2D**). A “module-trait” correlation heatmap presented the correlation between each module and clinical traits including immune clusters, survival time and status, and tumor stage. The yellow module had the strongest correlation with immune clusters (r = 0.78, **Figure 1F**). Furthermore, the close association between gene significance (GS) and module membership (MM) was addressed. A total of 256 hub mRNAs were subsequently identified under the cutoff: GS > 0.5 and MM > 0.6 (**Figure 1G**). ORA showed that immune-related biological processes were mainly enriched by these hub mRNAs (**Figure S2E**).

### 3.3 IRS construction and evaluation

Before model development, we performed univariate Cox analysis and identified 26 prognosis-related genes from the 256 hub mRNAs. The 26 mRNAs were subsequently subjected to the machine learning-based integrative procedure for identifying the optimal IRS with the highest accuracy and stability (**Table S4**).

**Figure 2:**
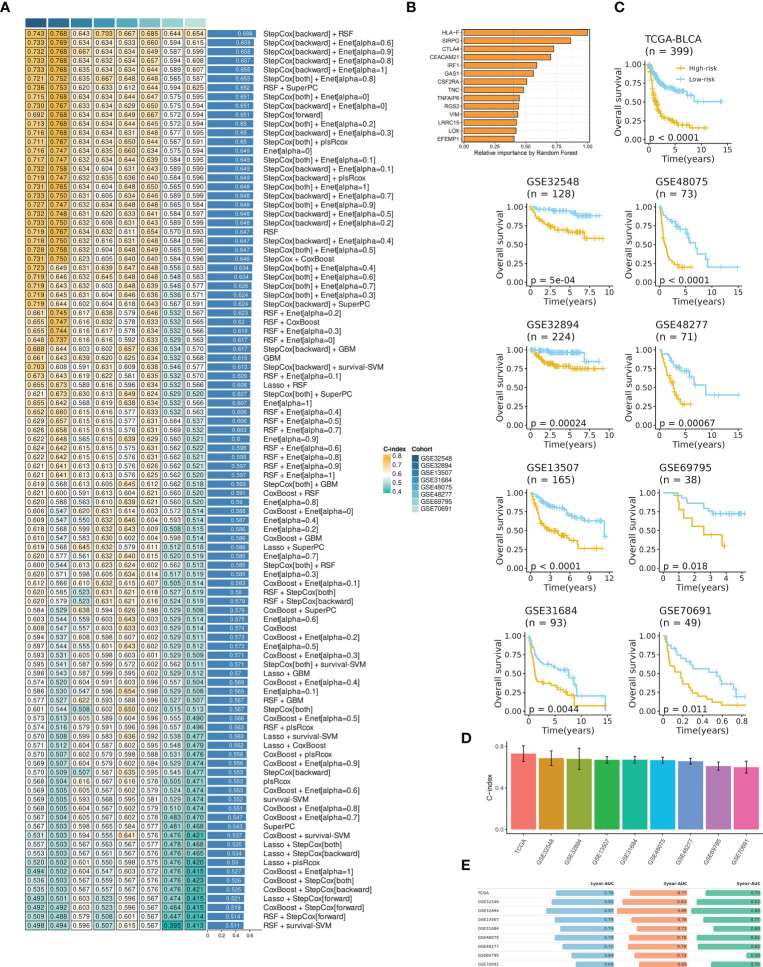
IRS development and evaluation in TCGA-BLCA and multiple GEO datasets. **(A)** The IRS was developed using a total of 101 machine-learning combinations *via* a 10-fold cross-validation framework. The c-index of each combination was evaluated in all datasets (training: TCGA-BLCA, and validation: GEO datasets). **(B)** The optimal model with the highest C-index was developed *via* a combination of backward StepCox and RSF. **(C)** High IRS was related to decreased overall survival across all datasets. Survival analysis was employed by Kaplan-Meier curves with the log-rank test. **(D)** The C-index of IRS across all datasets. **(E)** Time-dependent ROC analysis revealed the AUC of IRS for 1-, 3-, and 5-year OS. The table was visualized by formattable package and the length of each bar was correlated with value.

A final IRS with the best performance across several datasets was developed based on the combined StepCox (backward direction) and RSF (**Figure 2A**; **Table S5**). The IRS contained fourteen mRNAs (**Figure 2B**). BCa patients were divided into high- and low- IRS groups based on the median IRS score. BCa patients with high IRS had decreased survival time in all datasets (**Figure 2C**). The C-index of each cohort was presented in **Figure 2D**. Time-dependent ROC analysis was subsequently employed to measure the discrimination of IRS in terms of survival (**Figure 2E**). Collectively, results demonstrated that IRS had stable performance across multiple datasets.

**Figure 3:**
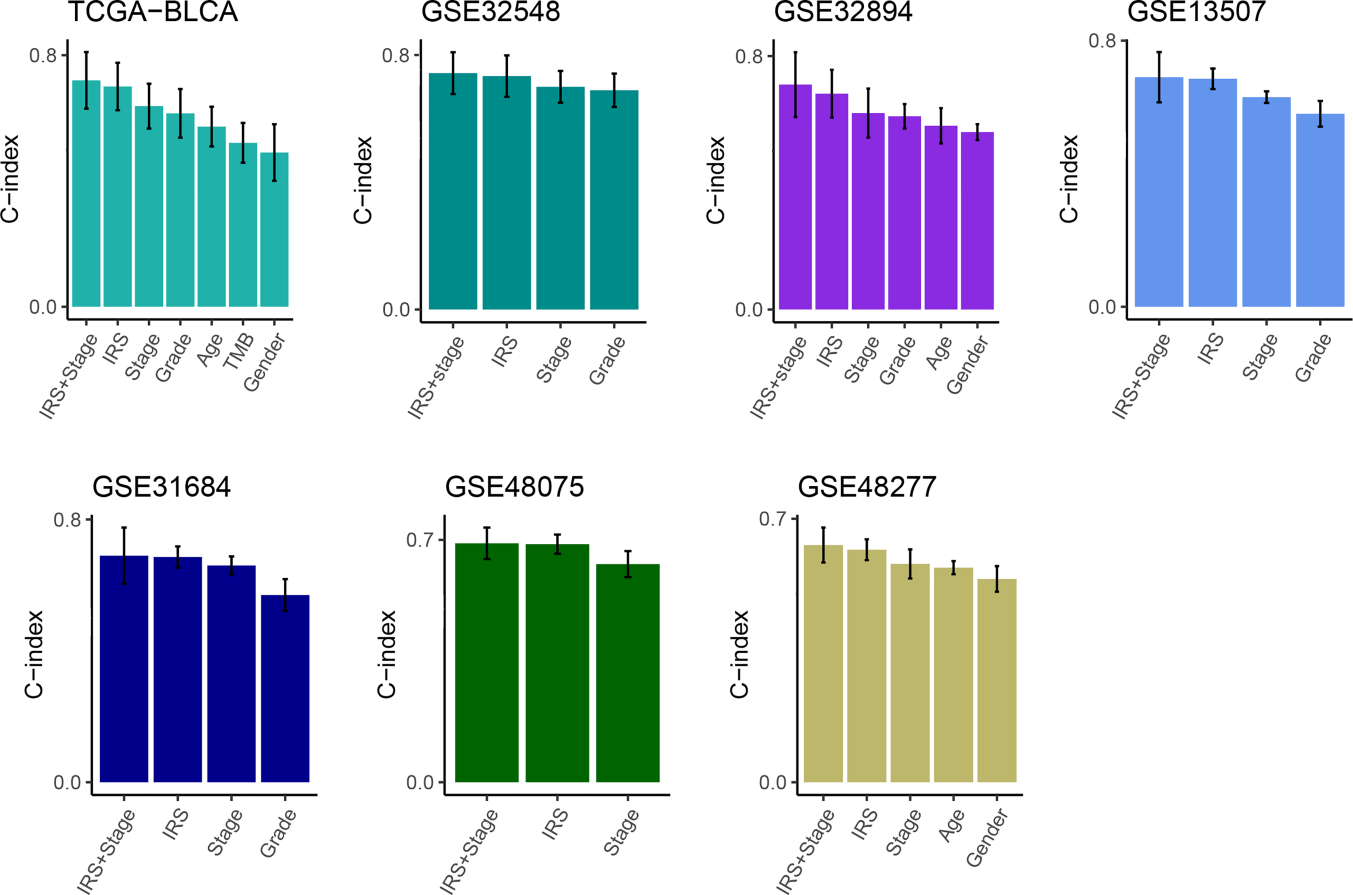
Comparison of the prognosis prediction performance of IRS with clinicopathological features and 94 published signatures. The C-index of IRS & stage, IRS, and clinicopathological features in TCGA-BLCA, GSE32548, GSE32894, GSE13507, GSE31684, GSE48075, and GSE48277.

Then, multivariate Cox analysis was performed in these datasets to figure out the possibility of IRS as an independent prognostic factor. Results showed that IRS and tumor stage were independent prognostic factors in multiple datasets (**Figure S3**). To further evaluate the superiority of the IRS over other clinicopathological features, we comprehensively compared the C-index between IRS and each feature and found that IRS performed well than others (**Figure 3**). Considering the wide application of tumor stage in practices for prognosis prediction, we combined the IRS and stage and found that the integrated model had a higher C-index compared to either IRS or stage alone (**Figure 3**).

### 3.4 Comparison of prognostic signatures

With rapid advances in high-throughput sequencing and bioinformatics, a growing number of predictive and prognostic signatures have been established in various tumors including BCa. In our study, we collected 94 prognostic mRNA signatures that were related to various biological features (e.g., necroptosis, pyroptosis, oxidative stress, and autophagy, **Table S6**). To compare the performance of the IRS between these published signatures, we calculated the C-index of each model. Remarkably, IRS displayed the highest performance in multiple datasets including TCGA, GSE32548, GSE32894, GSE13507, GSE31684, GSE48075, and GSE48277. In GSE69795 and GSE70691, IRS still showed higher performance than most published signatures (**Figure S4**).

### 3.5 IRS indicated decreased survival in the PUMCH_Uro cohort

In the era of translational medicine, we decided to validate the model constructed *in silicon* using an RNA-seq cohort from our single center.

**Figure 4:**
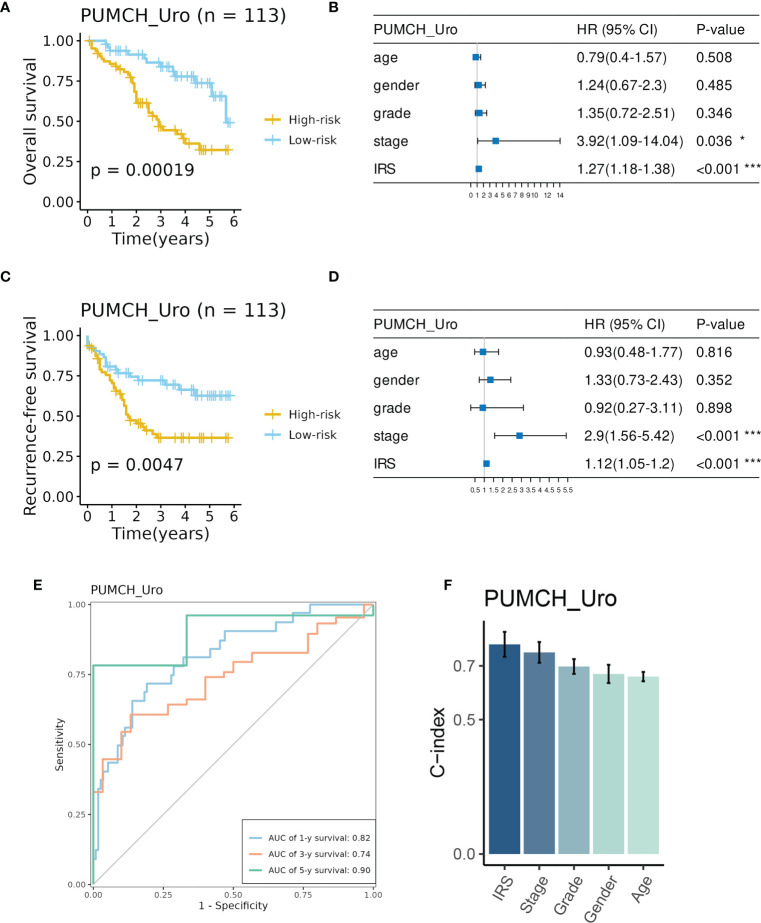
Evaluation of the prognosis prediction performance of IRS in the PUMCH_Uro cohort. **(A)** BCa patient in the High-risk group had decreased overall survival. **B** IRS and stage were independent risk factors for overall survival. **(C)** BCa patients in the high-risk group had decreased recurrence-free survival. **(D)** IRS and stage were independent risk factors for recurrence-free survival. **(E)** Time-dependent ROC curves of 1-, 3-, and 5-year overall survival. **(F)** The C-index of IRS and clinicopathological features. *p < 0.05; ***p < 0.001.

Consistently, high IRS indicated decreased survival time in terms of overall survival (OS, **Figure 4A**) and recurrence-free survival (RFS, **Figure 4C**). And IRS was regarded as an independent prognostic factor for OS and RFS by multivariate Cox analysis (**Figures 4B, D**). The time-dependent ROC analysis showed AUC of the 1-, 3-, and 5-year OS was 0.82, 0.74, and 0.9, respectively (**Figure 4E**). C-index also demonstrated the superiority of the IRS to other features (**Figure 4F**). These indicators showed the good and stable performance of the IRS in our in-house cohort.

### 3.6 IRS sculptured an inflamed and immunosuppressive TME of BCa

**Figure 5:**
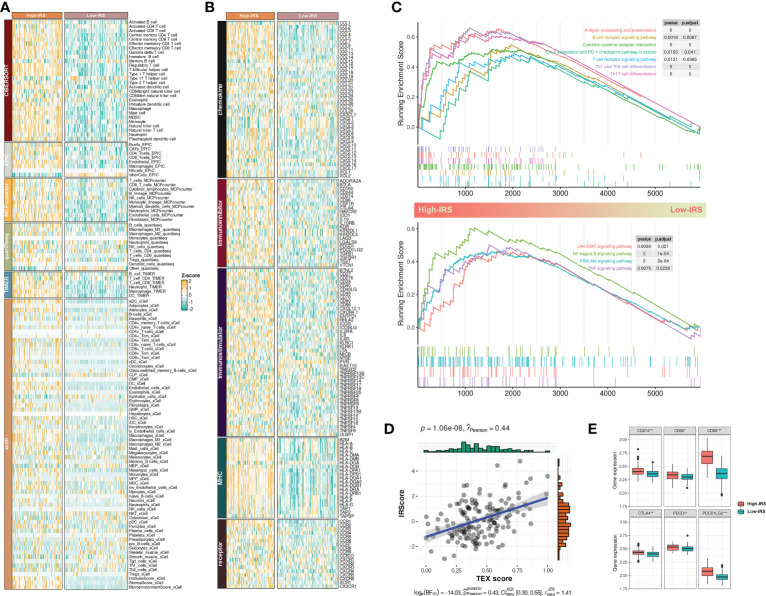
IRS sculptured an inflamed and immunosuppressive TME of BCa. **(A)** The differences in infiltration profiles of multiple immune cells between two clusters. The infiltration profiles were evaluated by several algorithms including CIBERSORT, EPIC, MCP-counter, quanTIseq, TIMER, and xCell, and then normalized and scaled into Z-score. **(B)** The differences in expression profiles of immune regulators between two clusters. The list of immune regulators was downloaded from TISIDB (http://cis.hku.hk/TISIDB/). **(C)** Upregulated biological processes and pathways in the High-IRS group. clusterProfiler package was used to perform the enrichment analysis. **(D)** Pearson Correlation between IRS and TEX signature score that calculated by ssGSEA. **(E)** The differences in expression profiles of immune checkpoints between two groups. P values were determined by Student’s t-test. *p < 0.05; ***p < 0.001.

As mentioned previously, we evaluated the infiltration abundances of various immune cells in the PUMCH_Uro cohort by several TME contexture decoding algorithms. TME of BCa in the high-IRS group was characterized by significantly higher infiltration levels of various immune cells compared to that in the low-IRS group (**Figure 5A**). Additionally, the expression profiles of immune regulators demonstrated a similar trend (**Figure 5B**). Similar results have been obtained by analyzing TCGA-BLCA (**Figures S5A, B**). Thorsson et al. (19) classified the pan-cancer TME into six immune subtypes. As expected, C4 (lymphocyte depleted) was related to dramatically lower IRS compared to other subtypes (**Figure S5C**).

Next, we investigated the dysregulated immune-related biological process and pathways between two groups by GSEA. Results showed that antigen processing and presentation, T/B cell receptor signaling pathway, PD-1/PD-L1 checkpoint pathway, cytokine-cytokine receptor interaction, and T helper cell differentiation were mainly upregulated in the High-IRS group (**Figure 5C**, up). Besides, conventional pathways including JAK-STAT, NK-kappa B, PI3K-AKT, and TNF were highly regulated in this group (**Figure 5C**, bottom). Overall, IRS may sculpt an inflamed TME of BCa.

A previous study reported that most infiltrated CD8+ T cells were termed “exhausted” as a consequence of complicated intercellular crosstalk (20). Exhausted T cells highly expressed multiple inhibitory receptors whereas losing potent effector functions. Besides, a pan-cancer T-exhausted cells-related signature has been reported in a previous study (21). In our study, we found a positive correlation between IRS and the T-exhausted cells-related signature (r = 0.44, **Figure 5D**). High-IRS group also had overexpressed inhibitory receptors including CD274, CD80, CD86, CTLA4, PDCD1, and PDCD1LG2 (**Figure 5E**). Hence, we speculated that high IRS indicated an immunosuppressive TME of BCa.

Collectively, these indicators suggested that IRS was related to an inflamed and immunosuppressive TME of BCa.

### 3.7 IRS predicted immunotherapeutic response in PUMCH_Uro

Although a large number of infiltrated CD8+ T cells have a change in phenotype, the inhibited immunity could be restored once we have revitalized these exhausted T cells. Theoretically, tumors with upregulated immune checkpoints (e.g., PD-L1) should be more sensitive to ICI treatment. That was the reason that motivated Food and Drug Administration to grant accelerated approval to atezolizumab for adult patients with unresectable locally advanced or metastatic triple-negative breast cancer whose tumors express PD-L1 (22). Similar results have been obtained by analyzing TCGA-BLCA (**Figure S5D**).

**Figure 6:**
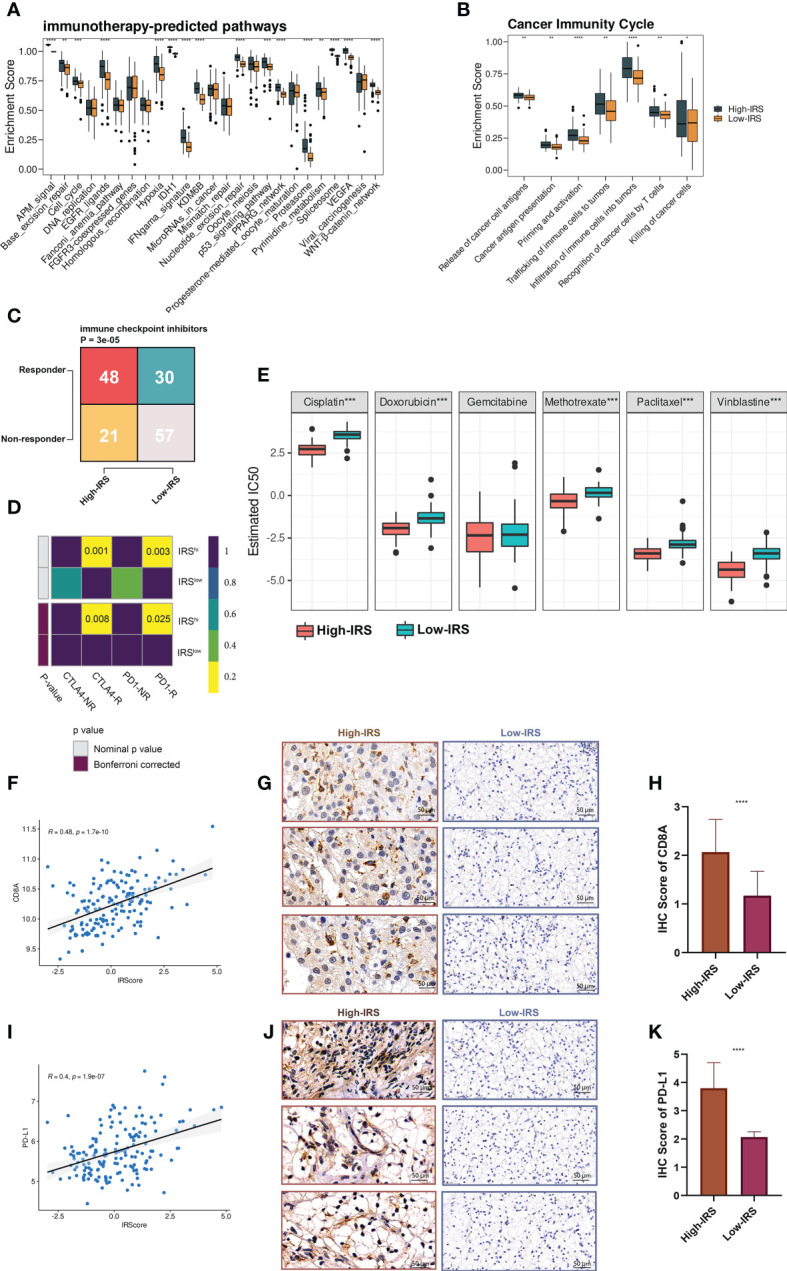
IRS predicted immunotherapeutic response in PUMCH_Uro. **(A)** The distribution of enrichment scores of several immunotherapy-predicted pathways between two groups. P values were determined by Student’s t-test. **(B)** The distribution of enrichment scores of seven steps in the anti-cancer immunity cycle between two groups. P values were determined by Student’s t-test. **(C)** The numbers of responders and non-responders between two groups based on the TIDE algorithm. P value was determined by the Chi-squared test. **(D)** SubMap module of GenePattern predicted the responses to anti-PD-1 and anti-CTLA-4 between the two groups. **(E)** The difference of estimated IC50 of six chemotherapy agents between the two groups. **(F)** Scatter plot demonstrated the correlation between IRS and expression of CD8A. **(G)** Representative IHC staining images of CD8A in two groups. Scale bars: 50 μm. **(H)** The distribution of IHC scores of CD8A between two groups. IHC scores were evaluated by IHC staining. **(I)** Scatter plot demonstrated the correlation between IRS and the expression of PD-L1. **(J)** Representative IHC staining images of PD-L1 in two groups. Scale bars: 50 μm. **(K)** The distribution of IHC scores of PD-L1 between two groups. Negative and positive controls for CD8A and PD-L1IHC staining were provided in **Figure S7**. *p < 0.05; **p < 0.01; ***p < 0.001; ****p < 0.0001.

In our study, we first explored the enrichment scores of several immunotherapy-predicted pathways and cancer immunity cycles between the High- and low-IRS groups. Interestingly, most pathways were overactivated in the high-IRS group (**Figure 6A**) and the cancer immunity cycles showed a similar trend (**Figure 6B**). Similar results have been obtained by analyzing TCGA-BLCA (**Figures S5E, F**).

Shirley Liu Lab developed a computational framework called TIDE (Tumor Immune Dysfunction and Exclusion), to evaluate the immunotherapeutic responses from the gene expression profiles of cancer samples (23). Based on the online framework, we procured the number of predicted responders and non-responders of our in-house cohort. Pearson’s chi-squared test showed that the high-IRS group had significantly more responders compared to the low-IRS group (**Figure 6C**). To verify the prediction robustness, the Subclass Mapping (SubMap) analysis of the GenePattern was performed. Results showed that high IRS was related to anti-CTLA-4 and anti-PD-1 immunotherapy responses (**Figure 6D**). Subsequently, Pearson correlation analysis uncovered the positive correlation between IRS and CD8A (r = 0.48, **Figure 6F**) and PD-L1 (r = 0.4, **Figure 6I**). IHC images (**Figures 6G, J**) and scores (**Figures 6H, K**) further proved that the protein expressions of CD8A and PD-L1 were remarkably higher in the high-IRS group. Collectively, BCa patients in the high-IRS group could benefit from immunotherapy more than those in the low-IRS group.

In addition, high IRS was predicted to be more sensitive to commonly used chemotherapy agents (except for gemcitabine, **Figure 6E**).

### 3.8 IRS predicted immunotherapeutic response in multiple ICI-treated cohorts

**Figure 7:**
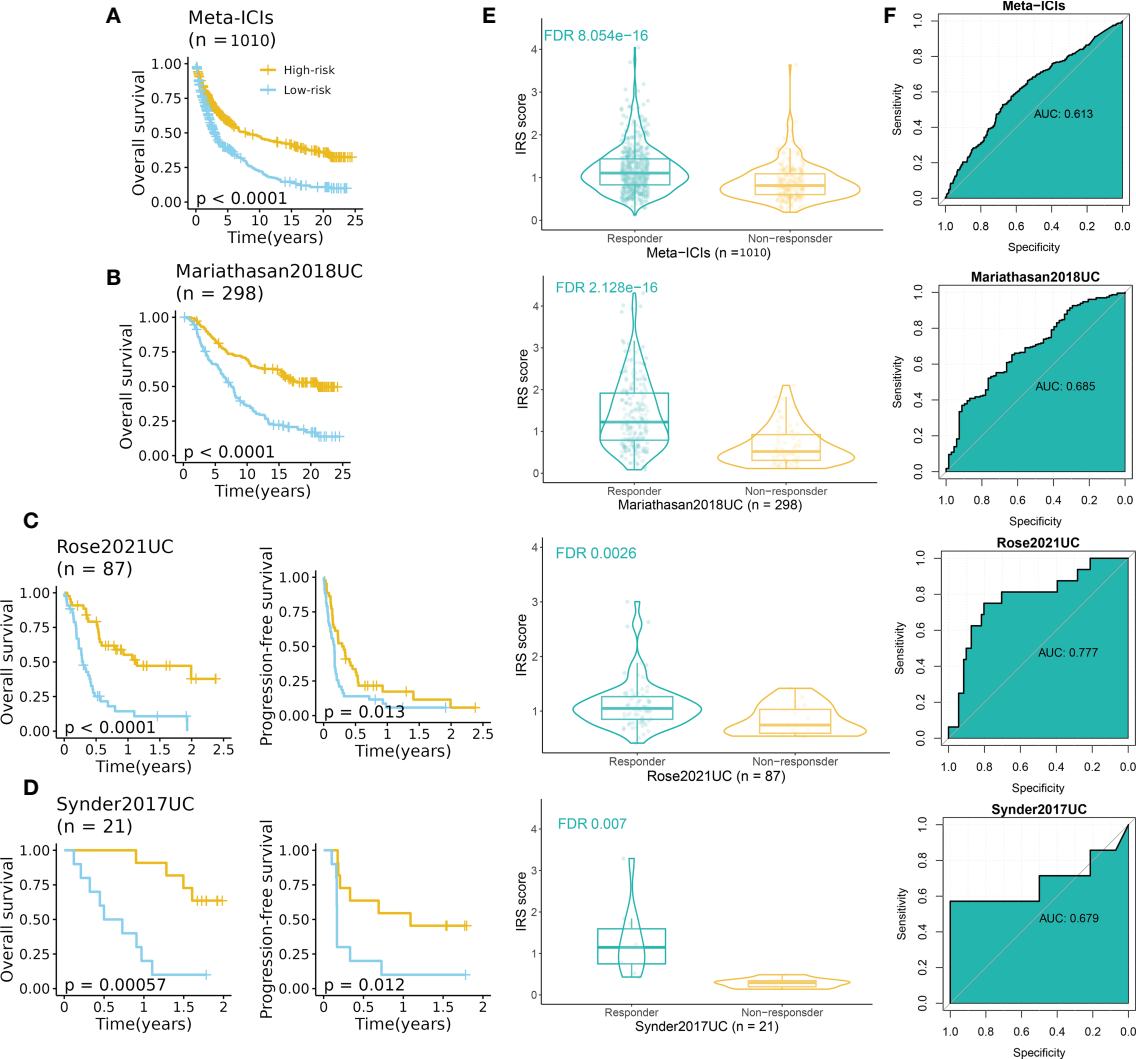
IRS predicted immunotherapeutic response in ICI cohorts. **(A)** High-IRS group was related to favorable overall survival in the ICI-meta cohort. **(B)** High-IRS group was related to favorable overall survival in the Mariathasan2018UC cohort. **(C)** High-IRS group was related to favorable overall survival in the Rose2021UC cohort. **(D)** High-IRS group was related to favorable overall survival in the Synder2017UC cohort. **(E)** Responders had remarkably higher IRS compared to non-responders. **(F)** ROC curves displayed the performance of response prediction of IRS in the above cohorts.

To further explore the predictive performance of IRS in terms of ICI response, we normalized the 13 ICI RNA-Seq cohorts into one GEO-meta cohort by removing batch effects as previously reported (24). Intriguingly, patients of high-IRS in the GEO-meta cohort had a prolonged survival time (**Figure 7A**). And responders had a strikingly higher IRS compared to non-responders (**Figure 7E**). The ROC analysis showed a sufficient predictive performance of the IRS (**Figure 7F**).

We then assess the performance of IRS in independent cohorts including UC, RCC, SKCM, NSCLC, and GC (no survival data). Expectedly, consistent results were observed in all cohorts (**Figures 7B–D**, **S6A–C**). In three UC cohorts, patients with high IRS had favorable prognoses in terms of overall survival and progression-free survival (**Figures 7B–D**). The AUC varied from 0.679 to 0.777. For other cancer types, the AUC varied from 0.601 (Hugo2016SKCM) to 0.759 (Riaz2017SKCM) (**Figure S6C**).

## 4 Discussion

ICI therapy has revolutionized treatment for advanced tumors whereas a limited population benefits from this treatment (25). Thus, screening novel biomarkers is urgently needed to address this concern. With the development of high-throughput sequencing and increased demand for precision therapy, a wealth of predictive and prognostic biomarkers have been identified, and mounting multigene signatures have been established in previous studies (26, 27). Although these published signatures demonstrated satisfactory performance in their training and external testing datasets, their performance decreased dramatically in other datasets (15). The limitation makes it necessary to fit a consensus signature for stratifying patients and to validate the performance across a large number of cohorts.

Tumor-infiltrating immune cells and tumor cells are major components of the TME, and intricate communication networks can regulate tumor development. Although immune cells play a critical role against cancer, malignant cells can in turn establish a very intricate balance in which distinct immune subtypes may boost tumor progression, metastasis, and drug resistance (28). This profound evidence motivated us to find a novel signature with high accuracy and stable performance, based on immune subtypes.

In our study, we first identified two immune subtypes based on the infiltration profiles of 28 immune cells *via* a robust clustering method. Two immune subtypes indicated distinct immunological patterns that cluster 1 (C1) was related to a “cold” TME whereas cluster 2 (C2) dominated an “inflamed” TME. WGCNA was employed to identify hub genes that had the strongest correlation with C2. After filtering genes unrelated to prognosis, we subjected the left genes to our well-designed, integrative pipeline to develop a consensus IRS with fourteen immune-related genes based on the combination of backward StepCox and RSF. The IRS demonstrated stable performance in predicting prognosis and was considered an independent prognostic factor in these datasets. Besides, thanks to the integrative framework in model construction, our IRS presented remarkable superiority in predicting prognosis to a majority of previously published models. Considering BCa patients of the high-IRS group suffering from poor prognosis, early identification of these patients and providing personalized treatment should extend the overall survival, indicating the appealing application of the IRS in the clinical setting.

In our in-house cohort, IRS performed well in discriminating overall survival and recurrence-free survival, that is to say, patients with high IRS had decreased survival. Additionally, both IRS and stage were independent factors for OS and RFS whereas IRS had a higher C-index than the stage. Interestingly, we noticed the superior performance of the tumor stage to IRS in predicting prognosis across many independent datasets including our in-house cohort. However, multivariate Cox analysis (**Figure S3**) showed that the IRS was more stable in predicting survival than the tumor stage, which failed to predict prognosis in GSE31684 and GSE48075. This confirmed the robustness of our developed prognostic model using an integrated machine-learning framework. Besides, considering the prognostic value of the tumor stage, its combination with our developed IRS can improve the performance in prognosis prediction. A series of *in-silicon analyses* and IHC revealed that high IRS was related to the inflamed and immunosuppressive TME of BCa. Promisingly, highly expressed inhibitory factors in the high IRS group predicted improved anti-cancer immunity and sensitivity to ICIs. Therefore, we speculated that the application ICIs to these patients should restore the inhibited anti-cancer immunity and prolong survival.

To verify our hypothesis, we next explore the performance of IRS in terms of prognosis and response to immunotherapy in multiple ICI-treated datasets. As expected, patients with high IRS benefited from both overall survival and progression-free survival. And the IRS was significantly higher in responders compared to that in non-responders. All these indicators suggested that our IRS performed well in prognosis and immunotherapeutic response prediction.

In our in-house cohort, we also explored the association between IRS and chemotherapy agents that were commonly used in urological practices. Results suggested that patients of the high-IRS group also benefited from chemotherapy drugs except for gemcitabine. The finding may provide theoretical fundamentals for immunotherapy combinations with chemotherapy for BCa patients with high IRS. Immunotherapy combinations with chemotherapy (namely “chemoimmunotherapy”) can improve the response rate and benefit patients in terms of OS and PFS, as reported by Galsky et al. in their IMvigor130 cohort (29). Consistent results have also been addressed in the JAVELIN Bladder 100 study where patients receiving chemoimmunotherapy achieved a 31% OS and 38% PFS benefit (30). Compared to ICI alone, the addition of chemotherapy can release large amounts of tumor antigen and boost the effect of effector lymphocytes (31). However, we should bear in mind that the improved response rate and prolonged survival may be at the cost of increased morbidity of adverse effects (32). Urologists and oncologists should balance the benefits and harms of combination therapy for BCa patients of high IRS.

There are some limitations in the study, although the clinical application of IRS in BCa is appealing. First, the datasets involved in this study were retrospectively designed. The IRS should be further validated in prospective, well-designed cohorts with complete clinicopathological features and survival information. Second, the biological roles of the fourteen genes were not comprehensively explored. Thus, further mechanical experiments are needed to unveil their functions.

## 5 Conclusions

Benefiting from the development of bioinformatics and technologies, we developed a consensus immune-related signature, IRS, based on the integrated machine learning program. IRS demonstrated robust performance in predicting prognosis and response to immunotherapy and chemotherapy across TCGA-BLCA, multiple GEO datasets, and pan-cancer ICI cohorts. These indicators suggested the promising application of IRS in urological practices for the early identification of high-risk patients and potential candidates for ICI application to prolong survival.

## Data availability statement

The datasets presented in this study can be found in online repositories. The names of the repository/repositories and accession number(s) can be found below: https://www.ncbi.nlm.nih.gov/geo/, GSE32894; https://www.ncbi.nlm.nih.gov/geo/, GSE13507; https://www.ncbi.nlm.nih.gov/geo/, GSE32548; https://www.ncbi.nlm.nih.gov/geo/, GSE31684; https://www.ncbi.nlm.nih.gov/geo/, GSE48075; https://www.ncbi.nlm.nih.gov/geo/, GSE48277; https://www.ncbi.nlm.nih.gov/geo/, GSE70691; https://www.ncbi.nlm.nih.gov/geo/, GSE69795; https://portal.gdc.cancer.gov/, TCGA-BLCA.

## Ethics statement

The studies involving human participants were reviewed and approved by the Institutional Review Board of Peking Union Medical College Hospital (PUMCH). The patients/participants provided their written informed consent to participate in this study.

## Author contributions

Conceptualization, HC, and ZJ; methodology, HC; software, HC; validation, HC; formal analysis, HC; investigation, HC; resources, WY; data curation, WY; writing—original draft preparation, HC; writing—review and editing, HC; visualization, HC; supervision, WY; project administration, ZJ. All authors have read and agreed to the published version of the manuscript.

## Supplementary material

Supplementary Methods The details of parameter tuning and model construction.
