# Supplementary material for: Machine learning-based identification of tumor-infiltrating immune cell-associated model with appealing implications in improving prognosis and immunotherapy response in bladder cancer patients
Source: Front Immunol. 2023 Mar 31;14:1171420. doi: 10.3389/fimmu.2023.1171420 (PMC10102422; doi:10.3389/fimmu.2023.1171420)
Supplement: Supplementary Methods — The details of parameter tuning and model construction. [file DataSheet_1.pdf]

Supplementary Material

Machine learning-based identification of tumor-infiltrating immune cell-associated model with appealing implications in improving prognosis and immunotherapy response in bladder cancer patients

Hualin Chen†, Wenjie Yang†, Zhigang Ji*

*** Correspondence:** Zhigang Ji jizhigang@pumch.cn

1 Supplementary Figures and Tables

1.1 Supplementary Figures

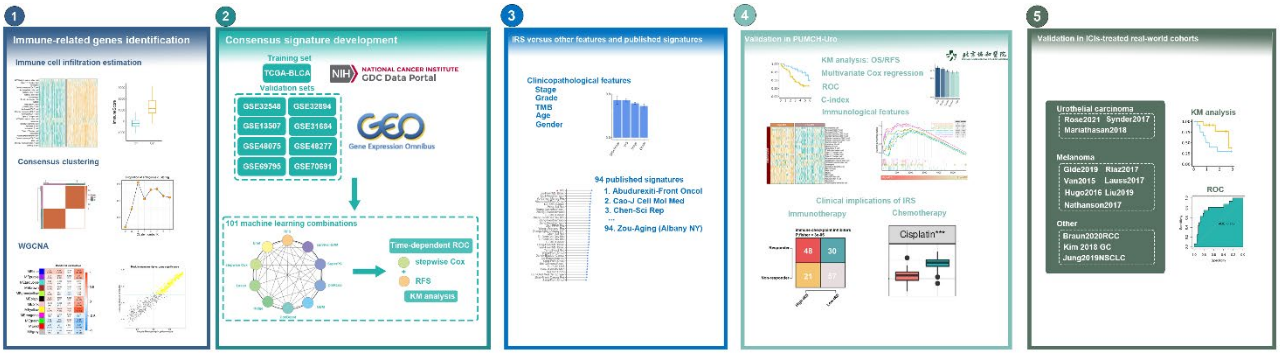

Figure S1 The overall design of the study.

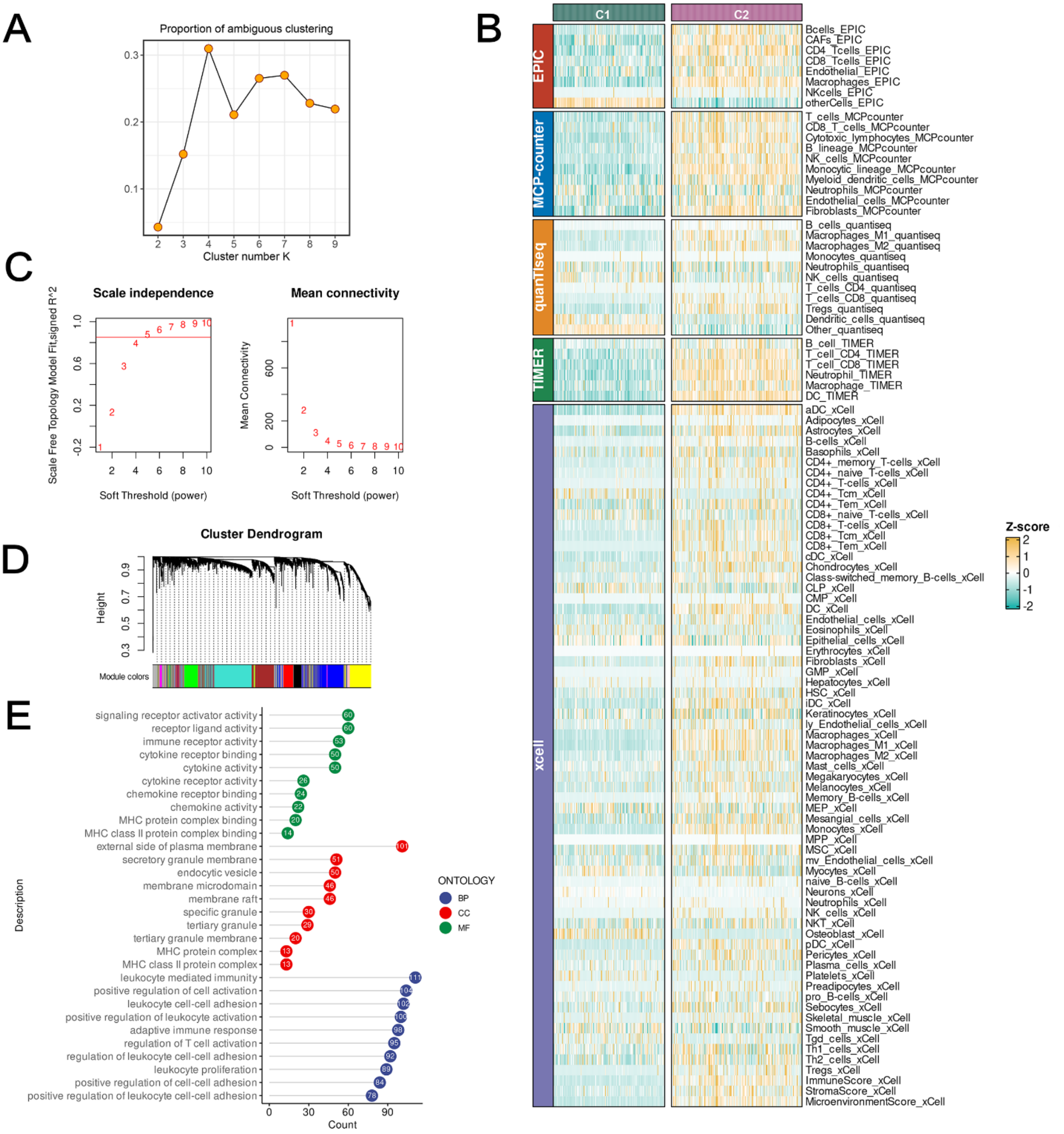

Figure S2 Unsupervised consensus clustering and WGCNA analysis. **A** The PAC scores of each k (cluster number). The lowest PAC suggested the optimal cluster number (k = 2). **B** The differences in infiltration profiles of multiple immune cells between two clusters. The infiltration profiles were evaluated by several algorithms including EPIC, MCP-counter, quanTIseq, TIMER, and xCell, and then normalized and scaled into Z-score. **C** Scale independence and mean connectivity determined the optimal sort threshold (power = 5). **D** Genes were clustered into several modules (indicated by each color) based on similarity in expression profiles. **E** GO enrichment analysis of hub genes of the yellow module.

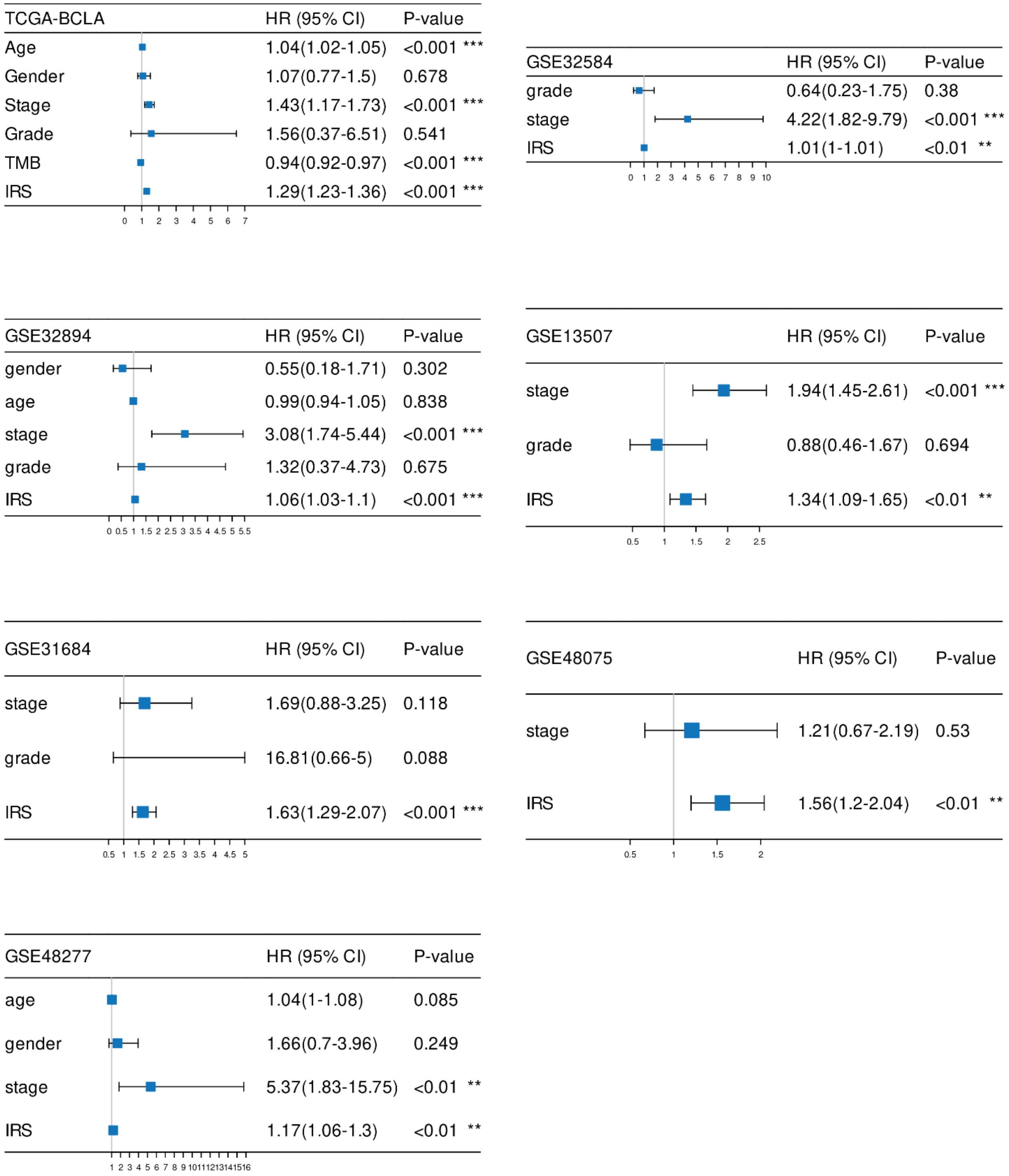

Figure S3 Forest plots demonstrated the independent risk factors for overall survival across multiple datasets based on multivariate Cox regression analysis.

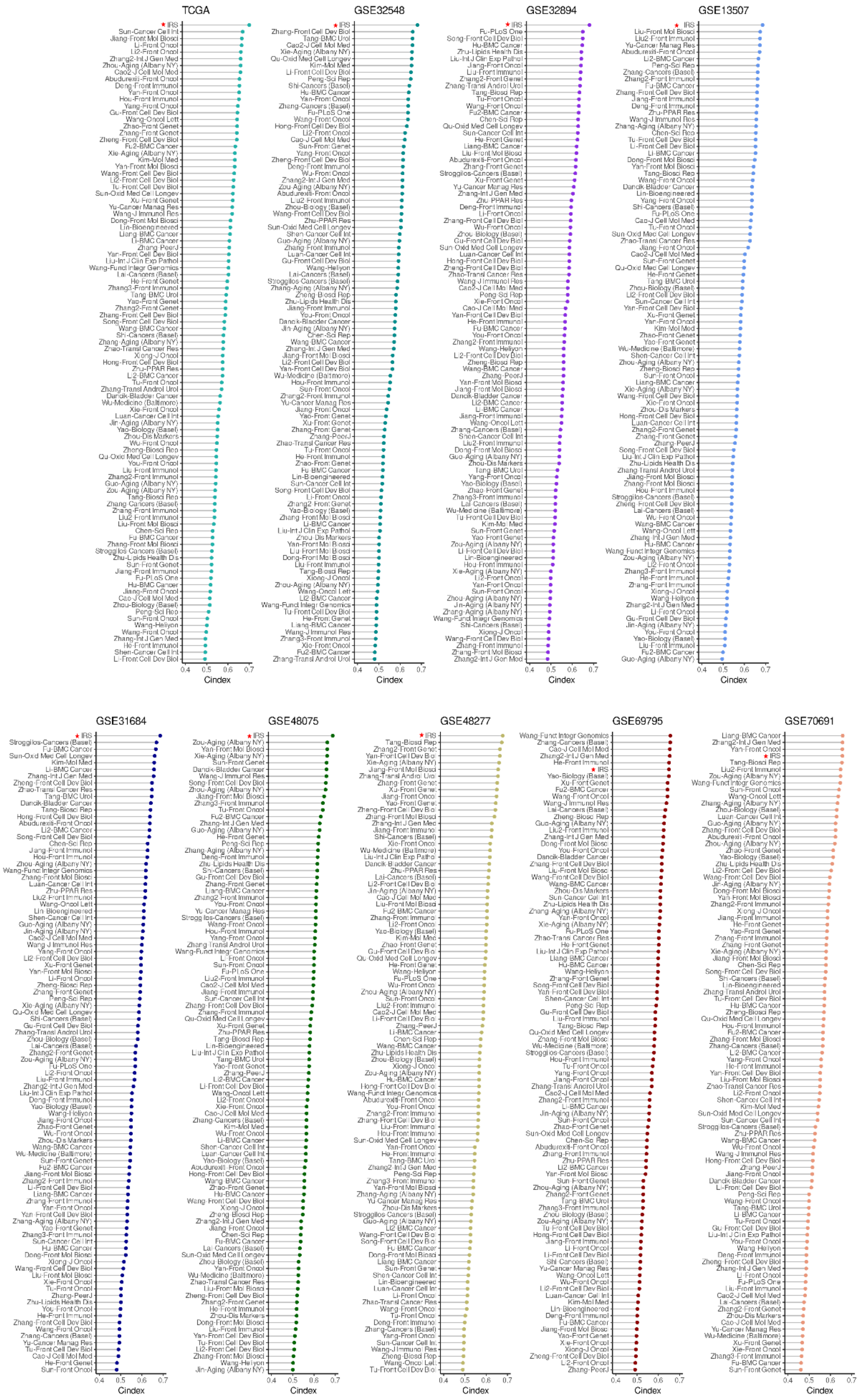
TCGA
GSE32548
GSE32894
GSE13507
GSE31684
GSE48075
GSE48277
GSE69795
GSE70691
IRS
Cindex
0.4
0.5
0.6
0.7

Figure S4 Comparison of the prognosis prediction performance of IRS with clinicopathological features and 94 published signatures. The C-index of IRS and 94 published signatures across all datasets.

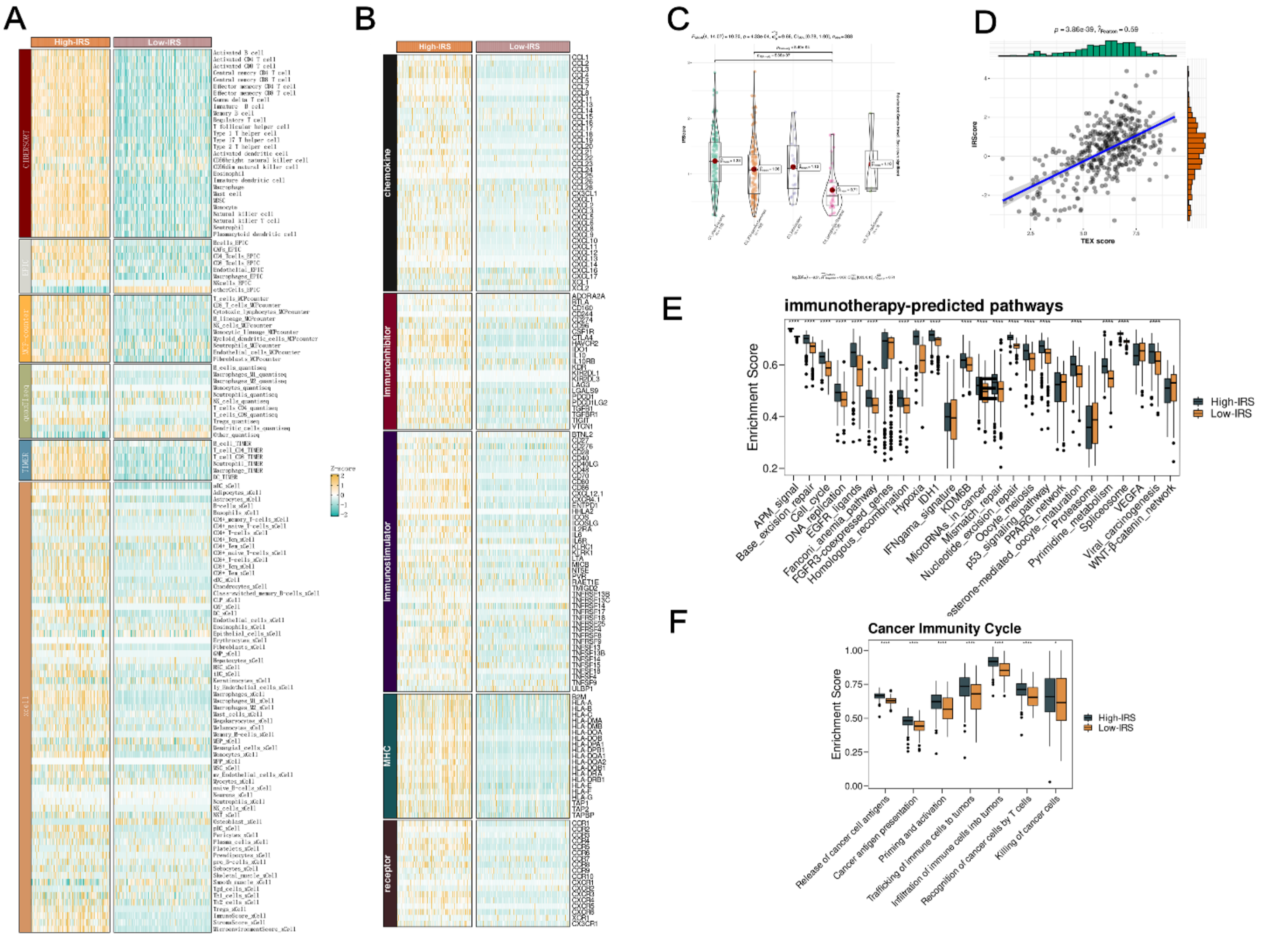

Figure S5 High IRS was related to an inflamed and immunosuppressive TME of BCa in the TCGA-BLCA dataset. **A** The differences in infiltration profiles of multiple immune cells between two clusters. The infiltration profiles were evaluated by several algorithms including CIBERSORT, EPIC, MCP-counter, quanTIseq, TIMER, and xCell, and then normalized and scaled into Z-score. **B** The differences in expression profiles of immune regulators between two clusters. **C** The distribution of IRS across five immune subtypes. **D** Scatter plot demonstrated the correlation between IRS and TEX signature score. **E** The distribution of enrichment scores of several immunotherapy-predicted pathways between two groups. **F** The distribution of enrichment scores of seven steps in the anti-cancer immunity cycle between two groups.

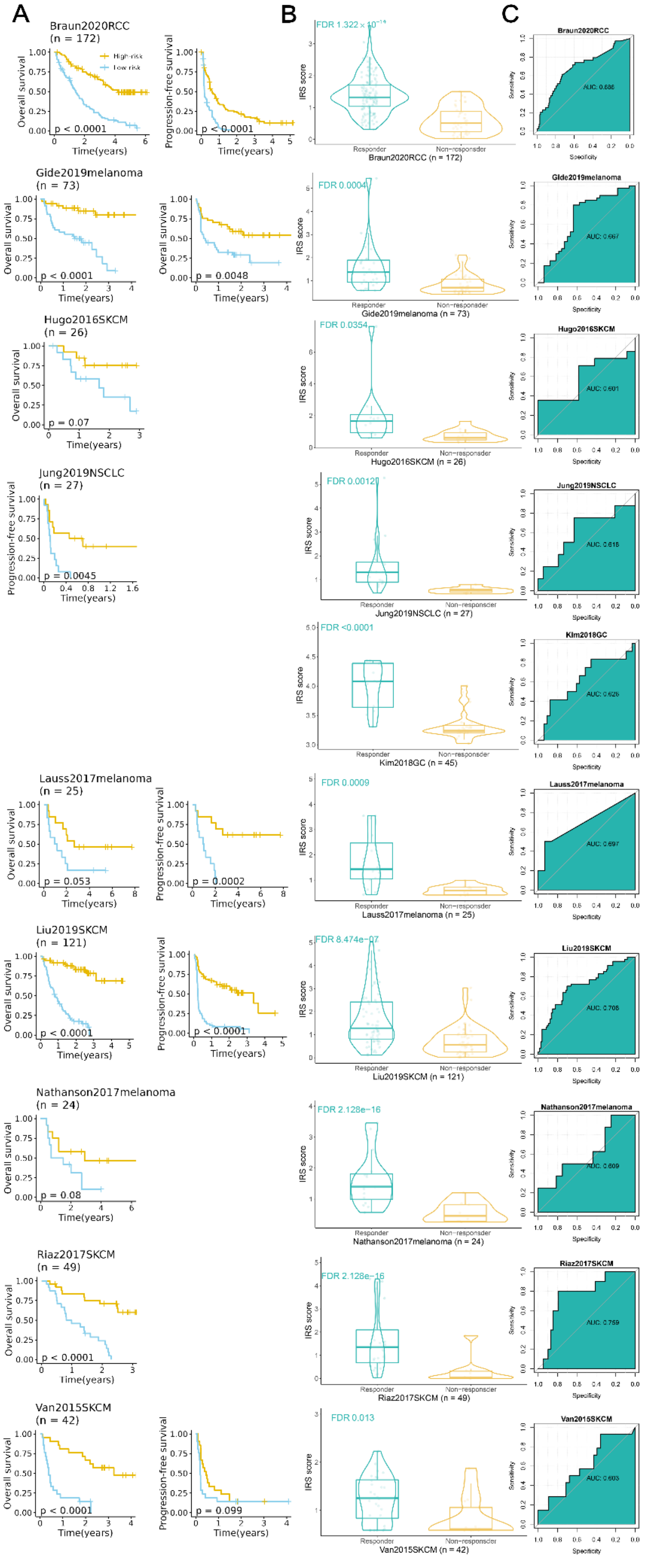
A
B
C
Braun2020RCC (n = 172)
Gide2019melanoma (n = 73)
Hugo2016SKCM (n = 26)
Jung2019NSCLC (n = 27)
Kim2018GC (n = 45)
Lauss2017melanoma (n = 25)
Liu2019SKCM (n = 121)
Nathanson2017melanoma (n = 24)
Riaz2017SKCM (n = 49)
Van2015SKCM (n = 42)
Overall survival
Progression-free survival
Time(years)
High-risk
Low-risk
IRS score
Responder
Non-responder
Sensitivity
Specificity

Figure S6 IRS predicted immunotherapeutic response in ICI cohorts. **A** High-IRS group was related to favorable overall survival and progression-free survival. **B** Responders had remarkably higher IRS compared to non-responders. **C** ROC curves displayed the performance of response prediction of IRS in the above cohorts.

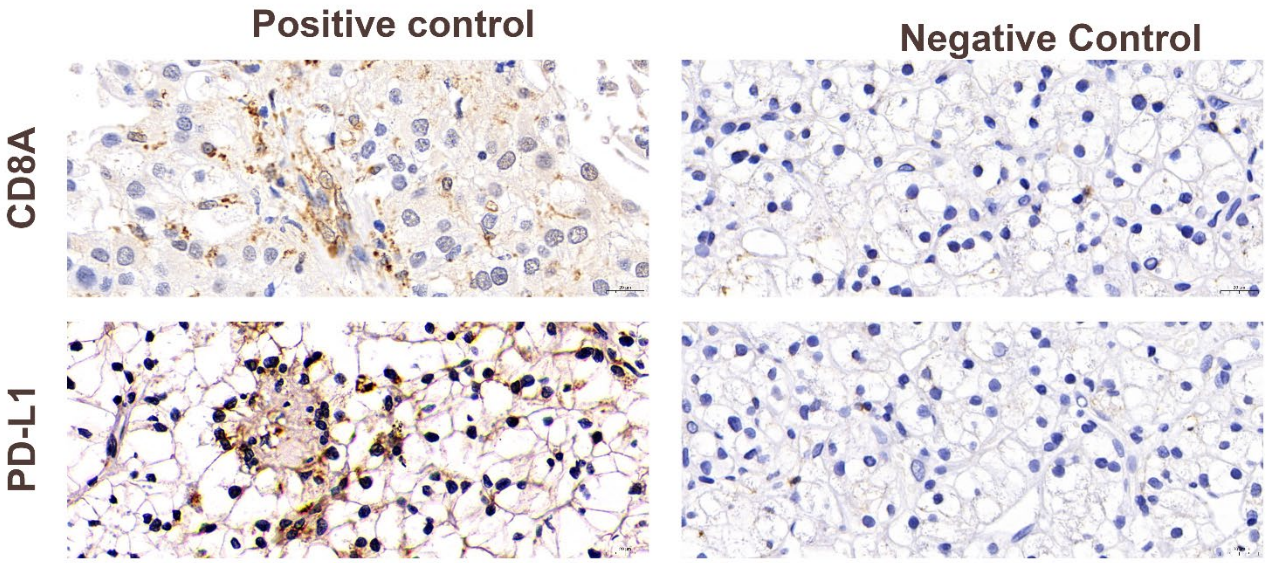

Figure S7 Positive and negative controls of IHC images for CD8A and PD-L1.

1.2 Supplementary Tables

Table S1: Characteristics of the inhouse PUMCH_Uro cohort, TCGA-BLCA, and GEO datasets

Table S2: Characteristics of ICIs cohorts

Table S3: The marker genes for 28 types of immune cells

Table S4: 26 prognosis-related mRNAs identified from the hub genes within the yellow module

Table S5: C-index of each model across all datasets

Table S6: 94 published signatures

1.3 Supplementary Methods

1.3.1 Machine learning

To develop a consensus machine learning-derived signature (IRS) with high accuracy and stability performance, we integrated 10 machine learning algorithms including random survival forest (RSF), elastic network (Enet), Lasso, Ridge, stepwise Cox, CoxBoost, partial least squares regression for Cox (plsRcox), supervised principal components (SuperPC), generalized boosted regression modeling (GBM), and survival support vector machine (survival-SVM). A few algorithms possessed the ability of feature selection, such as Lasso, stepwise Cox, CoxBoost, and RSF. Thus, we combined these algorithms to generate a consensus model. In total, 101 algorithm combinations were conducted on prognosis-related genes (identified by univariate Cox regression analysis) to fit prediction models based on 10-fold cross-validation. The initial signature discovery was performed in TCGA-BLCA. The RSF model was implemented via the *randomForestSRC* package. RSF had two parameters *ntree* and *mtry*, where *ntree* represented the number of trees in the forest and *mtry* was the number of randomly selected variables for splitting at each node. We used a grid-search on ntree and mtry using 10-fold cross-validation. All the pairs of (*ntree*, *mtry*) were formed and the one with the best C-index value was identified as the optimized parameters. The Enet, Lasso, and Ridge were implemented via the *glmnet* package. The regularization parameter, lambda, was determined by 10-fold cross-validation, whereas the L1-L2 trade-off parameter, α, was set to 0-1 (interval =0.1). The stepwise Cox model was implemented via s*urvival* package. A stepwise algorithm using the AIC (Akaike information criterion) was applied. The CoxBoost model was implemented via *CoxBoost* package, which is used to fit a Cox proportional hazards model by componentwise likelihood-based boosting. For the CoxBoost model, we used 10-fold cross-validation routine *optimCoxBoostPenalty* function to first determine the optimal penalty (amount of shrinkage). Once this parameter was determined, the other tuning parameter of the algorithm, namely, the number of boosting steps to perform, was selected via the function *cv.CoxBoost*. The dimension of the selected multivariate Cox model was finally set by the principal routine CoxBoost. The plsRcox model was implemented via *plsRcox* package. The *cv.plsRcox* function was used to determine the number of components requested, and the *plsRcox* function was applied to fit a partial least squares regression generalized linear model. The SuperPC model was implemented via *superpc* package, is a generalization of principal component analysis, which generates a linear combination of the features or variables of interest that capture the directions of largest variation in a dataset. The superpc.cv function used the10-fold cross validation to estimate the optimal feature threshold in supervised principal components. To avoid problems with fitting Cox models to small validation datasets, it uses the "pre-validation" approach.

The GBM model was implemented via the *gbm* package. Using the 10-fold cross validation, the *cv.gbm* function selected index for number trees with minimum cross-validation error. The *gbm* function was used to fit the generalized boosted regression model. The survival-SVM model was implemented via *survivalsvm* package. The regression approach takes censoring into account when formulating the inequality constraints of the support vector problem.

1.3.2 IRS construction

The IRS signature was established based on the combination of StepCox (backward direction) and RSF. StepCox algorithm was used to screen out the most valuable features. RSF algorithm was further used to filtrate the most reliable model. Log-rank score test for splitting survival trees was conducted. First, the x-variable x was assumed to be ordered as $x_1 \le x_2 \le \ldots \le x_n$. Then, the "ranks" for each survival time T_j ($j \in [1, \ldots, n]$) were computed. The obtained equation is as follows:

$$\mathbf{a}_{\mathbf{j}} = \delta_j - \sum_{k=1}^{\Gamma_j} \frac{\delta_k}{n - \Gamma_k + 1}$$

where $T_k = \#\left[t : \mathrm{T}_t \le \mathrm{T}_k\right]$ and T_j represents the index of the order for T_j. The log-rank score test came as follows:

$$\text{IRS signature} = \mathrm{S}\ (\mathrm{x}, \mathrm{c}) = \frac{\sum_{xk \le c}(a_j - n_l \bar{a})}{\sqrt{n_l\left(1 - \frac{n_l}{n}\right) s_a^2}}$$

where $\bar{a}$ and S_a^2 represent the sample mean and sample variance of $[a_j : j = 1, \ldots, n]$, respectively. The measure of node separation is determined using log-rank score splitting by | S (x, c) |. The best split is reached by maximizing this value over x and c.
